# The ineligibility of food products from across the EU for marketing to children according to two EU-level nutrient profile models

**DOI:** 10.1371/journal.pone.0213512

**Published:** 2019-10-23

**Authors:** Stefan Storcksdieck genannt Bonsmann, Marguerite Robinson, Jan Wollgast, Sandra Caldeira

**Affiliations:** European Commission, Joint Research Centre (JRC), Ispra, Italy; Tabriz University of Medical Sciences, ISLAMIC REPUBLIC OF IRAN

## Abstract

**Background:**

A variety of nutrient profiling models have been developed to restrict food marketing to children. Previous assessments have shown substantial differences in terms of model strictness and agreement, but EU-wide data on how leading products in the various national markets perform against these health-minded nutrition criteria are unavailable.

**Objective:**

To evaluate the nutritional composition of the pre-packaged food offer in selected categories sold at scale in the EU using criteria of two nutrient profile models intended to restrict food marketing to children.

**Methods:**

The nutrient profile models of the private-sector EU Pledge and of the World Health Organization's Regional Office for Europe were applied to a commercial database with sales and nutritional information of 2691 pre-packaged products from five product categories (breakfast cereals, ready meals, processed meat, processed seafood, and yoghurts) and 20 EU countries. This study describes the criteria not met, the product ineligibility rates, and the distances to the various criteria thresholds.

**Findings:**

Between 48% (EU Pledge) and 68% (WHO Europe) of the 2691 products analysed were found to be ineligible for marketing to children. The criteria thresholds most often not met were those for total sugars (in breakfast cereals, yoghurts), salt (in processed meat, processed seafood, ready meals), and fibre (in breakfast cereals). Total and saturated fat criteria also played a substantial role in rendering yoghurt products ineligible, and the energy criterion did so for ready meals.

**Interpretation:**

A large number of food products selling at scale in the EU do not meet the criteria of two EU-level nutrient profile models intended to restrict food marketing to children. Given the considerable market share of many such products, they are likely to be consumed widely and in some cases regularly, including by children, even without being marketed to them. Nutrient profile models could serve as benchmarking tools for monitoring and evaluating food product reformulation efforts.

## Introduction

A nutritionally balanced diet is a cornerstone of good health. However, the high prevalence of overweight and obesity in many countries [1,2] indicates that people of all ages are not getting the balance right. Among the various information environment factors driving overweight, obesity, and diet-related diseases, the marketing of foods high in fat, sugars, and salt (HFSS) has been shown to be detrimental, specifically to children's diets [3,4]. For this reason, calls to reduce the HFSS food marketing pressure on children appear in many high-level policy documents [5–9]. Addressing this issue, the recently revised EU Audiovisual Media Services Directive states that "Member States should be encouraged to ensure that self- and co-regulation, including through codes of conduct, is used to effectively reduce the exposure of children to audiovisual commercial communications regarding foods and beverages that are high in salt, sugars, fat, saturated fats or trans-fatty acids or that otherwise do not fit those national or international nutritional guidelines” [10]. In addition, as overconsumption of energy-rich, nutrient-poor foods is a well-established risk factor for the development of non-communicable diseases (NCDs) [11], other initiatives are ongoing across the globe to promote a more healthful food supply in the first place and thus facilitate healthier diets for all age groups.

Nutrient profiling is used to classify or rank foods "according to their nutritional composition for reasons related to preventing disease and promoting health" [12]. Systematic approaches have been proposed for developing nutrient profile models that include decisions about whether to use category-specific or across-the-board systems, the selection of nutrients and reference bases, and what specific thresholds to set [13,14]. Nutrient profile models have been developed for various purposes including the regulation of food advertising and marketing to children. Moreover, studies have shown that nutrient profile models can be used to assess diet quality, and that poorer ratings are associated with increased risk of various NCDs [15–18]. In this study, we report the results of applying two nutrient profile models to pre-packaged products from 20 EU countries in the food categories of breakfast cereals, processed meat, processed seafood, ready meals, and yoghurts. We chose the nutrient profile models of WHO Europe [12] and the EU Pledge [19] as European-level models, devised to restrict food marketing and advertising to children. Our previous comparison of the criteria between these models indicated that the WHO nutrient profile model is stricter than the EU Pledge in the majority of nutrient thresholds [20]. However, concrete data on product eligibility from across Europe remain scarce [21] despite its importance as more and more countries develop codes of conduct to address food marketing pressure and will likely need to select a nutrient profile model in the process.

This study shows the proportion of products deemed ineligible for marketing to children by category under the two models as well as the corresponding nutrients of concern. It also describes the amount by which products in the different categories fail to meet the applicable criteria thresholds of the WHO Europe model.

## Methods

### Euromonitor product database characteristics

Euromonitor International (www.euromonitor.com) is a commercial global market research company, providing data on the packaged food industry. Their Nutrition database covers 20 of the EU28 countries. The database contains the following nutrition information: energy, carbohydrates, sugars, fibre, protein, salt, fat, and saturated fat, all per 100 g or 100 ml of product. The database tracks private and branded products sold through retail establishments (e.g. supermarkets, small grocery stores, and online retailers), with a focus on the leading market products. The Packaged Food database tracks market sales for 25 of the 28 EU countries. It is important to note that products with multiple variants (e.g., nut, fruit, or chocolate variants of breakfast cereals) or flavours (e.g., yoghurts flavoured with strawberry, raspberry, pear) are aggregated to form a single data entry to which a market share is assigned, with a representative product chosen to assign the nutrient content to the group. Euromonitor does not currently allow the identification of the representative flavour or variant chosen, nor does it allow identification of products whose market shares are aggregated from those that are not.

### Choice of nutrient profile models

The focus of our assessment is on the European Union. Consequently, we chose the nutrient profile models of the EU Pledge (hereafter EU Pledge model) [19] and the WHO Regional Office for Europe (hereafter WHO Europe model) [12] as these models have a matching Europe-wide scope. Notably, the WHO Europe model has also already been used in defining school food procurement standards in Malta and to assess product healthfulness in general [22,23], and nutrient profile systems with this express purpose show similar criteria to the models of the EU Pledge and WHO Europe.

### Product category selection

The primary condition for selecting product categories was that comparable product category definitions existed in the EU Pledge and WHO Europe models as well as the Euromonitor product database (see Table 1 for details). In addition, nutrition information for all corresponding nutrient profile model criteria had to be complete in the Euromonitor database to allow for a full assessment under both models. Five relevant and comparable categories emerged: breakfast cereals, processed meat, processed seafood, ready meals, and yoghurts. Other food categories such as chocolate, sweet biscuits, cakes, and sugar-sweetened beverages are not included in the analysis because either the WHO Europe model (for chocolate, sweet biscuits, cakes) or both models (for sugar-sweetened beverages) do not specify any criteria but rather exclude them entirely from advertising to children by default.

**Table 1:** Product categories selected for inclusion in the nutrient profile comparison, their corresponding categories in the Euromonitor database, and the nutrient profile categories in the EU Pledge and WHO Europe models.

Product category	Euromonitor category	EU Pledge category	WHO Europe category	Comments
Breakfast cereals	Breakfast cereals (subcategories: hot cereals, children's breakfast cereals, flakes, muesli, other ready-to-eat cereals)	Category 6C (ready-to-eat breakfast cereals such as cornflakes, puffed rice, porridge) Criteria Energy: ≤210 kcal/portion Sodium: ≤450 mg/100 g Saturated fats: ≤5 g/100 g Total sugars ≤30 g/100 g Fibre: ≥3 g/100 g	Category 6 (oatmeal; cornflakes; chocolate breakfast cereals; mueslis) Criteria: Total fat: ≤10 g/100 g Total sugars: ≤15 g/100 g Salt: ≤1.6 g/100 g Fibre: >6 g/100 g	EU Pledge portion for breakfast cereals ranges from 30 g to 45 g; while calculations have been made for both portion sizes, only the results for a 30 g portion are reported here. In the WHO Europe model, it is at a country's discretion to include a threshold for minimum dietary fibre content, and WHO Europe proposes the value stated here.
Processed meat	Processed meat (subcategories frozen, chilled, shelf stable)	Category 3 (meat-based products: all kinds of processed meat/poultry, and meat products, consisting of minimally 50 g of meat per 100 g finished product) Criteria Energy: ≤170 kcal/portion Sodium: ≤800 mg/100 g Saturated fats: ≤6 g/100 g Total sugars: (≤5 g/100 g) Protein: ≥12% of energy	Category 14 (processed meat, poultry, fish and similar) Criteria Total fat: ≤20 g/100 g Salt: ≤1.7 g/100 g	EU Pledge portion for processed meat ranges from 45 g to 125 g; while calculations have been made for both portion sizes, only the results for a 45 g portion are reported here.
Processed seafood	Processed seafood (subcategories frozen, chilled, shelf stable)	Category 4 (fishery products: all kinds of processed fish, crustaceans and shellfish, consisting of min. 50 g of fish, crustaceans, and/or molluscs per 100 g of finished product) Criteria Energy: ≤170 kcal/portion (>170 kcal/portion if ≥25% total fat is PUFA) Sodium: ≤450 mg/100 g Saturated fats: ≤33% of total fat is SAFA (including TFA) Total sugars: (≤5 g/100 g) Protein: ≥12% of energy	Category 14 (processed meat, poultry, fish and similar) Criteria Total fat: ≤20 g/100 g Salt: ≤1.7 g/100 g	EU Pledge portion for processed seafood ranges from 45 g to 125 g; while calculations have been made for both portion sizes, only the results for a 45 g portion are reported here.
Ready meals	Ready meals (subcategories: shelf stable ready meals, chilled pizza, chilled ready meals, dried ready meals, frozen pizza, frozen ready meals, prepared salads)	Category 7B (composite dishes, main dishes, and filled sandwiches: all kinds of dishes & sandwiches containing min. 2 of the following: 30 g fruit, vegetables, cereals, meat, fish, milk or any combination of those (calculated as fresh equivalent) per portion. (Thresholds apply to food as reconstituted, ready for consumption, following manufacturer’s instructions) Criteria Energy: ≤425 kcal/portion Sodium: ≤400 mg/100 g Saturated fats: ≤5 g/100 g Total sugars: ≤7.5 g/100 g	Category 9 (ready-made and convenience foods and composite dishes) Criteria Energy: ≤225 kcal Total fat: ≤10 g/100 g Saturated fat: ≤4 g/100 g Total sugars: ≤10 g/100 g Salt: ≤1 g/100 g	
Yoghurts	Yoghurt (subcategories: drinking yoghurt, plain yoghurt, fruited yoghurt, flavoured yoghurt)	Category 5A (dairy products other than cheeses: must contain minimum 50% dairy (Codex Alimentarius standard)) Criteria Energy: ≤170 kcal/portion Sodium: ≤300 g/100 g Saturated fat: ≤2.6 g/100 g Total sugars: ≤13.5 g/100 g Protein: ≥12 E% or ≥2 g/100 g or 100 ml	Category 7 (yoghurts, sour milk, cream and other similar foods) Criteria Total fat: ≤2.5 g/100 g Saturated fat: ≤2 g/100 g Total sugars: ≤10 g/100 g Salt: ≤0.2 g/100 g	EU Pledge portion for yoghurts was set at 150 g based on a stated size range of 150–200 ml and a note that portions differed widely due to diversity of products.

NB: The WHO Europe nutrient profile model also specifies the following generic criteria thresholds: ≤ 1 g per 100 g total fat in the form of industrially-produced trans fatty acids, < 0.5% of total energy in the form of alcohol. These criteria could not be considered in our analysis owing to unavailability of such information in the Euromonitor Nutrition database. Likewise, not all criteria specified in the EU Pledge model could be considered owing to unavailability of data in the Euromonitor Nutrition database. For example, the EU Pledge specifies different energy thresholds for processed seafood products depending on the content of polyunsaturated fatty acids. For yoghurts, the EU Pledge sets minimum levels for calcium, vitamin D or any B vitamin. None of these nutrients is covered in the Euromonitor Nutrition database. Abbreviations: SAFA, saturated fats; TFA, trans fats

### Extraction of product information from Euromonitor database

All results presented herein are based on data extracted from Euromonitor. Nutrient content per 100 g or 100 ml, representing the situation in 2015, was extracted for all categories listed in Table 1. Brand market shares were also extracted. Only the 20 EU countries tracked in the Nutrition 2016 database were included.

A list of products for each country and category was generated as follows:

1. A list of products selling in 2015 was generated from the brand share data.
2. For each product in the list, a search was performed in the nutrition dataset for a product with an identical brand name and category. If the product was located, the full available nutrition information was extracted.
3. A check was then performed for the presence of data for each criterion included in the two nutrient profile models. If the relevant data was missing for any criterion, that product was removed from the list. For the food categories considered in this analysis, fibre is only a required nutrient in the category of breakfast cereals. For all other categories, where fibre content was missing, a default value of 0 g fibre per 100 g (or 100 ml) was recorded.
4. A few products had highly implausible energy values; a consistency check was applied to remove products with profiles not satisfying the relationship:

4×protein[g]+4×carbohydrates[g]+2×fibre[g]+9×fat[g]=energy[kcal]±50

The factors are based on energy values defined in EU Regulation 1169/2011 (18), bearing in mind that the Euromonitor product database does not hold information on other calorific ingredients such as organic acids or polyols. A safety factor of ±50 kcal was included in the calculation as a reasonable figure to rule out the exclusion of products with minor data inaccuracy while acknowledging the crudeness of the calculation. The cost of applying this consistency check is that products which have all the correct data for the required nutrients may potentially be removed if they have an incorrect value recorded in the database for another nutrient. The percentage of products removed due to the consistency check ranged from 1% (yoghurts) to 6% (processed seafood).

### Application of nutrient profile model criteria

The extraction of products with complete nutrition criteria data and sensible calorie information resulted in a total of 2691 products for the five product categories (see Table 2). All products extracted were on the market in 2015, yet the quantities consumed and their marketing are not explicitly considered in the analysis presented hereafter. Given the small sample size at country level, no comparison between individual countries is attempted here. Disaggregated country level data is nonetheless provided as supporting information (Tables A-B in S1 File).

**Table 2:** Number of products from the Euromonitor Nutrition 2016 database with complete nutrition criteria information and non-zero brand share by category and country.

Country^a^	Breakfast cereals	Processed meat	Processed seafood	Ready meals	Yoghurts	TOTAL
AT	22	34	24	34	25	139
BE	42	26	13	43	35	159
BG	20	8	3	4	17	52
CZ	37	12	11	18	47	125
DK	35	22	15	11	13	96
FI	39	42	11	30	49	171
FR	41	32	20	36	30	159
DE	37	30	17	42	42	168
GR	27	11	11	32	37	118
HU	45	31	15	11	44	146
IE	53	11	9	19	38	130
IT	26	38	27	43	51	185
NL	22	26	16	23	38	125
PL	48	7	12	23	32	122
PT	40	20	18	20	36	134
RO	19	2	1	4	17	43
SK	33	6	10	18	29	96
ES	27	24	26	32	35	144
SE	51	51	29	28	20	179
GB	57	37	20	47	39	200
TOTAL	721	470	308	518	674	2691
Mean±SD	36±11	24±14	15±7	26±13	34±11	135±41
Minimum	19	2	1	4	13	43
Maximum	57	51	29	47	51	200

Product numbers are the result of extracting all products from the Euromonitor Nutrition 2016 database with complete and plausible nutrition information as well as a non-zero brand share as indicated in the Euromonitor Packaged Foods 2016 database. SD, standard deviation.

^a^ ISO 3166–1 alpha-2 country code.

The full set of criteria in each model and product category (see Table 1) was applied to compute the number and percentage of products that were ineligible for marketing to children. Furthermore, all individual criteria (energy, nutrients) were applied separately to elucidate the impact of individual thresholds within the various categories.

### Estimation of reformulation needed for product eligibility

In order to understand the compositional change required for ineligible products in each category to pass the applicable criteria thresholds of both nutrient profile models, we computed the minimum, median, and maximum distance from the respective thresholds. Due to space constraints, only the WHO Europe model figures are discussed here; the EU Pledge figures are reported in the Supplementary Information.

### Statistical analyses

Descriptive statistics were computed using Microsoft Excel 2010. Differences between the two models, in terms of the percentage of products not meeting the corresponding criteria, were assessed using two-tailed, homoscedastic Student's t-test, with a significance level of p < 0.05.

## Results

### Ineligible products under the two nutrient profile models

Across all product categories and countries, the EU Pledge model classified 48% (1281) of the 2691 products analysed as ineligible for advertising to children, ranging from 29% for yoghurts to 65% for processed meat (Table 3). The corresponding figures for the WHO Europe model were 68% (1822) for all products, 31% for the smallest share (processed seafood), and 80% for the largest share (breakfast cereals). Differences between models are statistically significant for breakfast cereals (*p*<0.0001), processed seafood (*p* = 0.0014), yoghurts (*p*<0.0001), and for the total number of ineligible products (*p*<0.0001).

**Table 3:** Number and percentage of products from the Euromonitor Nutrition 2016 database not passing the EU Pledge and WHO Europe nutrient profile model criteria, respectively, by category and overall.

	Breakfast cereals	Processed meat	Processed seafood	Ready meals	Yoghurts	Total
EU Pledge ineligible						
# of products	267	306	183	329	196	1281
% of total	37%	65%	59%	64%	29%	48%
WHO Europe ineligible						
# of products	574	304	96	334	513	1821
% of total	80%^a^	65%	31%^b^	64%	76%^a^	68%^a^

Statistically significant differences between EU Pledge and WHO Europe model:

^a^ p < 0.0001

^b^ p = 0.0014. Product ineligibility was assessed for all products listed in the Euromonitor Nutrition 2016 database with complete and plausible nutrition information and non-zero brand share as indicated in the Packaged Foods 2016 database. For the EU Pledge model application, portion sizes had to be assumed for breakfast cereals (30 g, only for the energy criterion), ready meals (200 g, only for the energy criterion), yoghurts (150 g, only for the energy criterion) as well as for processed meat and processed seafood (both 45 g). For the WHO Europe model application, the recommended optional minimum fibre content of 6 g/100 g of product was included in assessing the breakfast cereals category.

In some instances, the same product is counted multiple times in the above figures, due to being sold in different countries, thus overestimating the number of different products analysed. However, we deem this acceptable as the product composition may differ between countries (and the comparison remains valid in any case, as all products are assessed with both models). To illustrate this point: at the generic brand name level the database showed 408 duplicates for breakfast cereals, whereas this number was reduced to 113 when all nutritional information was included in the automatic duplicate search. Removing these true duplicates affected the figures reported here only negligibly (+/- 1 percentage point or 0.1 g, respectively). For the other product categories, true duplicates were substantially lower at 4 (processed meat), 6 (processed seafood), 4 (ready meals), and 38 (yoghurts) products. Country-level data on product ineligibility rates can be found in the Supplementary Information.

### Most restrictive nutrient profile model criteria

Knowledge of the most restrictive nutrient profile model criteria–to be understood as rendering the highest number of products ineligible for marketing to children–helps to identify the nutrients of concern in each category and where product reformulation could make a major impact. It may also give an indication about products that are "out of reach" for reformulation. Based on the WHO Europe model and looking at the number of thresholds not met in each product category, we observed a downward trend from the percentage of products exceeding only one threshold to the percentage exceeding all thresholds (Table 4). In the categories with four or five applicable criteria, the majority of products did not meet one or two criteria. Still, 10–22% of ineligible products did not meet three criteria thresholds, and 13% of all ineligible ready meal products missed four of the five applicable criteria thresholds. It should be noted that thresholds for total sugars, total fat, saturated fat, and energy represent upper limits, whereas thresholds for protein and fibre represent the minimum levels to be achieved.

**Table 4:** Overview of number of products and their proportions per number of WHO Europe nutrient profile model criteria not met in each of the five product categories from the Euromonitor Nutrition 2016 database.

	Number of criteria thresholds not met					
	0	1	2	3	4	5
Breakfast cereals						
# products	147	265	253	55	1	n/a
% of total in this category (n = 721)	20%	37%	35%	8%	<1%	n/a
% of ineligible products (n = 574)	n/a	46%	44%	10%	<1%	n/a
Processed meat						
# products	166	232	72	n/a	n/a	n/a
% of total in this category (n = 470)	35%	49%	15%	n/a	n/a	n/a
% of ineligible products (n = 304)	n/a	76%	24%	n/a	n/a	n/a
Processed seafood						
# products	212	84	12	n/a	n/a	n/a
% of total in this category (n = 308)	69%	27%	4%	n/a	n/a	n/a
% of ineligible products (n = 96)	n/a	88%	13%	n/a	n/a	n/a
Ready meals						
# products	184	148	69	73	43	1
% of total in this category (n = 518)	36%	29%	13%	14%	8%	<1%
% of ineligible products (n = 334)	n/a	44%	21%	22%	13%	<1%
Yoghurts						
# products	161	209	196	102	6	n/a
% of total in this category (n = 674)	24%	31%	29%	15%	1%	n/a
% of ineligible products (n = 514)	n/a	40%	39%	20%	1%	n/a

Product ineligibility under the WHO Europe model was assessed for all products listed in the Euromonitor Nutrition 2016 database with complete and plausible nutrition information and non-zero brand share as indicated in the Packaged Foods 2016 database. For breakfast cereals, thresholds are specified for total sugars, total fat, salt, and fibre. For processed meat and processed seafood, total fat and salt thresholds apply. For ready meals, the following five criteria apply: total fat, saturated fat, total sugars, salt, and energy. Finally, four criteria applied to the yoghurt category: total fat, saturated fat, total sugars, and salt. The criteria were applied individually and then the number of thresholds not complied with counted. Percentages were calculated relative to the total number of products in the respective category and relative to the number of ineligible products. n/a, not applicable.

Across all countries and product categories, the total sugars criterion removed the largest number of products, namely 839 products, which corresponds to 31% of all products and to 44% of all products to which a total sugars threshold applies (breakfast cereals, ready meals, and yoghurts). The median excess of total sugars relative to the WHO Europe model threshold was 19% for ready meals, 30% for yoghurts, and 67% for breakfast cereals, with some products containing three times the limit of 15 g/100 g (Table 5).

**Table 5:** Assessment of the minimum, median, and maximum difference from applicable nutrient/energy thresholds in five product categories from the Euromonitor Nutrition 2016 database using the WHO Europe nutrient profile model.

	Breakfast cereals (721 products)				Processed meat (470 products)		Processed seafood (308 products)		Ready meals (518 products)					Yoghurts (674 products)			
Criterion	Total sugars	Salt	Total fat	Fibre	Total fat	Salt	Total fat	Salt	Total fat	Saturated fat	Total sugars	Salt	Energy	Total fat	Saturated fat	Total sugars	Salt
Threshold per 100 g	≤15 g	≤1.6 g	≤10 g	≥6 g	≤20 g	≤1.7 g	≤20 g	≤1.7 g	≤10 g	≤4 g	≤10 g	≤1.0 g	≤225 kcal	≤2.5 g	≤2 g	≤10 g	≤0.2 g
Not meeting threshold (n)	452	57	113	318	104	272	30	78	112	113	8	268	181	326	196	379	30
Not meeting threshold (%)	63%	8%	16%	44%	22%	58%	10%	25%	22%	22%	2%	52%	35%	48%	29%	56%	4%
Difference from threshold:																	
Minimum	0.3 g	0.1 g	0.1 g	0.1 g	0.1 g	0.10 g	0.7 g	0.1 g	0.1 g	0.1 g	1.0 g	0.1 g	1 kcal	0.1 g	0.1 g	0.1 g	0.1 g
Median	10.0 g	0.4 g	6.0 g	2.5 g	5.0 g	0.5 g	12.5 g	1.3 g	2.5 g	0.9 g	1.9 g	0.4 g	36 kcal	0.9 g	0.5 g	3.0 g	0.1 g
IQR	6.0–14.0 g	0.2–0.4 g	4.0–8.0 g	1.2–3.5 g	3.0–11.8 g	0.3–0.9 g	3.6–18.0 g	0.6–2.3 g	1.4–4.0 g	0.4–1.7 g	1.0–3.7 g	0.2–0.7 g	18–112 kcal	0.5–1.8 g	0.3–2.2 g	2.0–4.0 g	0.1–0.2 g
Maximum	29.4 g	1.2 g	15.3 g	6.0 g	36.8 g	4.3 g	38.0 g	14.2 g	27.1 g	8.0 g	6.0 g	12.0 g	305 kcal	7.9 g	5.1 g	18.4 g	1.1 g

NB: Note that thresholds for total sugars, total fat, saturated fat, and energy represent upper limits, whereas the threshold for fibre is a minimum level to be achieved. Furthermore, as for the percentages of products not meeting applicable thresholds, individual products may fail to comply with multiple criteria. Differences from the various applicable criteria thresholds were assessed for all products listed in the Euromonitor Nutrition 2016 database with complete and plausible nutrition information and non-zero brand share as indicated in the Packaged Foods 2016 database. For the upper limit values (total sugars, salt, total fat, saturated fat, and energy), the threshold figure was subtracted from the actual figures in the nutrition information. For the lower limit value (fibre), the actual figures in the nutrition information were subtracted from the threshold figure. Resulting values above zero were used to calculate the minimum, median, interquartile range (IQR), and maximum differences from the applicable category thresholds. Corresponding data for the EU Pledge model can be found in Tables C-E in S1 File.

This was followed by salt (705 products, 26% of total) and total fat (685 products, 25%), for which thresholds apply in all five categories. The median salt excess ranged from 25% in breakfast cereals to 76% in processed seafood. Salt levels showed by far the most pronounced median excess in processed seafood, processed meat, and ready meals, and median excess total fat was highest in processed seafood, processed meat, and breakfast cereals (Table 5).

The saturated fat criterion removed 309 products, which corresponds to 11% of the total number of products and to 26% of the 1192 products to which this criterion applies (ready meals and yoghurts). Levels of excess were similar in the two categories relative to the respective threshold.

The fibre criterion applied only to breakfast cereals and removed 318 products, which makes 12% of the total and 44% of the breakfast cereals. According to the nutrition information in the Euromonitor database, 56 of these products contained 2 g or less fibre per 100 g of product, which corresponds to one third or less of the fibre level recommended in the WHO Europe model.

## Discussion

We assessed the ineligibility of pre-packaged food products across Europe for marketing to children, using the nutrient profile models of the EU Pledge and WHO Europe. Overall, between 48% (EU Pledge model) and 68% (WHO Europe model) of the 2691 products studied were ineligible for marketing to children, with substantial variation between categories (31–80% with the WHO Europe model, 29–65% with the EU Pledge model). In terms of sales volume, Euromonitor estimates that in 2015 alone, over 6.2 million tonnes of yoghurts and over 5.5 million tonnes of processed meat products were purchased in the 28 EU Member States. Relatively smaller, but still substantial, quantities of ready meals (3.9 million tonnes), processed seafood products (1.9 million tonnes) and breakfast cereals (1.3 million tonnes) were sold. Judging by these estimated retail sales volumes for the five product categories, the products assessed are purchased and consumed annually in large quantities within the European Union. While not representing true individual intakes, the sales figures are indicative of consumption levels within the population. These results show that a large number of food products sold at scale are too high in total sugars, salt, saturated fat, and total fat, and too low in fibre based on EU-level nutrient profile model criteria. While such criteria were developed for the sole purpose of restricting food marketing and advertising to children, they can also be considered nutrition and health-minded food composition references. To our knowledge, this is the first Europe-wide assessment, covering 20 EU Member States and applying two pan-European nutrient profile models.

### Product ineligibility rates

Around two-thirds of processed meat, processed seafood, and ready meals, and about one-third of breakfast cereals and yoghurts did not meet the nutrition criteria of the EU Pledge model. Of note, with the revised EU Pledge nutrition criteria [24], released in October 2018 and fully effective end of 2019, an additional 200 products would become ineligible, taking the total ineligibility rate to 55% (1492 products). Product categories affected are breakfast cereals (+55 products), processed seafood (+46 products), and yoghurts (+99 products), owing to changes in sugar and salt thresholds. The revised criteria may provide additional incentives for food manufacturers to improve the nutritional composition of products to be marketed from 2020 onwards, so that these products are eligible for marketing to children. With the WHO Europe model, breakfast cereals and yoghurts were the most critical categories with over three quarters of products being ineligible, followed by processed meat and ready meals at ca. two-thirds, and processed seafood at one-third. Previous studies have reported higher ineligibility percentages for the EU Pledge, namely 59% [25] and 74.4% [26]. For the WHO Europe model, similar or higher ineligibility percentages relative to our observation have been reported from Europe (68%) [21], Canada (70.2%) [27], New Zealand (71%) [28], and Mexico (83.1%) [29], respectively. Differences in product eligibility rates between studies using the same nutrient profile model are largely owing to the choice of product categories. Our selection of products was guided by the availability of comparable categories in the two nutrient profile models used as well as the Euromonitor database, combined with sufficient information about the products' nutritional composition to apply the nutrient profile model criteria. We excluded product categories for which one or both models did not specify nutrient criteria, such as soft drinks, chocolate, or biscuits, notwithstanding their potential negative impact on public health. As far as the differences between the WHO Europe model and the EU Pledge model are concerned, these mainly derive from the differences between criteria thresholds in both models [20]. Beyond these differences, a large number of the products analysed in our study, one half to two thirds, do not meet the nutrient profile model criteria used to define food and drinks products eligible for marketing to children. These products, albeit in many cases not children's products, are (or can be) consumed by children. Importantly, our analysis only covers products that sell at a sizeable scale, yet their rating in the nutrient profile models would suggest their abundant consumption to be incompatible with good long-term health.

Where products are close to the various criteria thresholds, reformulation may be possible to shift them into the eligible range. For products far from one or more nutrient thresholds, comparable products meeting those thresholds may indicate that reformulation is nonetheless feasible. However, some products may be difficult, if not impossible, to reformulate to meet the criteria. Whilst reformulation could still be used to improve intakes of specific nutrients in these cases, other tools such as fiscal measures or front-of-pack nutrition labelling (including clear portion guidance) could be considered as a means to improve food choices, including through reducing consumption frequencies or smaller portions. It has been suggested that nutrient profiling could have further use for those measures and more broadly throughout the whole food supply chain too, including as benchmarks for food procurement and the monitoring and evaluation of nutrition and health policies [30].

### Impact of individual criteria and required reformulation

Using the WHO Europe model, the total sugars criterion was the most restrictive for the categories breakfast cereals and yoghurts, rendering 63% and 56% of products ineligible, respectively. Salt, in turn, was most limiting for processed meat (58%), ready meals (52%), and processed seafood (25%). Together with the overall higher product exclusion rate compared to the EU Pledge model, this finding supports the statement by Wicks *et al*. [31] that typically the sugars and salt thresholds make nutrient profile models of public bodies stricter than those developed by industry. Close to half of all breakfast cereal products (44%) did not meet the optional fibre threshold, making it the second most restrictive criterion for this category. Given the popularity of breakfast cereals, this may be viewed as a missed opportunity for increasing consumers' fibre intake, which regularly falls short of intake recommendations [32]. Of note, children's breakfast cereals have particularly high mean total sugars content across countries (23.5–33.4 g/100 g) [33], meaning they are not fit for advertising to children under the WHO Europe model.

Assessments of salt intake of adults across Europe reveal a range of 7–13 g salt per capita per day [34], which is well in excess of the 5 g limit recommended by the WHO [35]. Importantly, it has been estimated that, in developed countries, some 75% of daily salt intake comes from processed foods [36,37]. Furthermore, recent analyses indicate that about 50–65% of total sugars intake in Europe is from added sugars [38,39], with the highest values being observed in school-age children. Other dietary survey data from across Europe indicate that children and adolescents on average exceed recommended intakes for total sugars and saturated fat while falling short of fibre recommendations [40]. For adults, excess saturated fat and insufficient fibre intakes were noted, with a more mixed picture regarding the intake of total sugars [41]. Sodium intakes also tended to be too high in these three age groups.

Against this backdrop, food product reformulation becomes a primary lever for adjusting the dietary supply of sugars, salt, and other nutrients of public health concern, which in turn should lead to more adequate intakes of these nutrients [42,43]. Regular analysis would help to see whether the food offer improved over time as producers commit to reducing salt, saturated fat, and added sugars. Such commitments form an essential part of the EU platform for action on diet, physical activity and health [44] and the EU Frame Framework for National Initiatives on Selected Nutrients [45], and an EU roadmap for action on food product improvement was agreed by public and private stakeholders in 2016 [46]. For reformulation to have a true impact on public health, it is important that meaningful compositional changes are achieved and that the products make a substantial contribution to people's diets. In this regard, nutrient profile models can be useful benchmarking tools to monitor and evaluate food reformulation efforts. The recently revised EU Pledge nutrition criteria indicate that less strict nutrient criteria could provide short- to mid-term targets towards meeting stricter criteria such as those of the WHO Europe model, which in turn might serve as long-term goals.

### Limitations

Some limitations apply to our study both in terms of the data used and the conclusions reached.

Since we do not have actual consumption statistics for the specific products assessed, it is not possible to make any direct correlations between their composition, population nutrient intakes, overall diets, and health. These restrictions are further compounded by the fact that diets are naturally expected to be composed of foods from more than the five categories considered here. However, the sales volume data give an indication about the relevance of these products in people's daily diets.

For some countries, the number of products in a given category was small (<10). Consequently, the ineligibility rates and theoretical reformulation required are less precise for these countries, especially where very few products are ineligible.

The EU Pledge and the WHO Europe nutrient profile model have as their aim the restriction of food advertising and marketing to children, respectively. As stated in the WHO Europe model, product lists for model testing should be compiled from products commonly eaten or frequently marketed to children (ideally both) [12]. While our database is not specific for products marketed to children, it does include products targeted at children and contains the leading pre-packaged products in the five categories. Moreover, our intention was not to validate the nutrient profile models but rather to assess popular products currently on the market using tested nutrient profile models. A previous study has used the WHO Europe model in a similar way to assess the healthfulness of the packaged food and beverage supply in India [22].

Since product category definitions differ to some degree between the Euromonitor database, the EU Pledge model, and the WHO Europe model, there is a risk that not all products were assessed with the appropriate category criteria. However, we sought to keep this risk to a minimum by limiting our choice of products from the Euromonitor database to those best matching the equivalent categories of the two nutrient profile models.

In case of multiple flavour variants of a specific product, Euromonitor chooses one product as representative (see Methods section for details). Should these flavour variants differ in nutritional composition, the product in the Euromonitor database might differ in terms of eligibility for marketing relative to one or more of the other flavour variants and thus create a bias in the results. However, this error is likely to be non-systematic and small as we would expect a cancelling out of products misclassified in one way or the other.

Last, due to lack of data in the Euromonitor Nutrition 2016 database, any nutrient profile model criteria concerning micronutrients, alcohol, or trans fats could not be considered.

The above notwithstanding, the Europe-wide scope of our assessment of products selling at scale in five relevant food product categories adds an important perspective to the debate around food reformulation, innovation, and diversification in the food supply.

## Conclusions

Excessive intakes of energy, saturated fats, sugars, and salt remain a priority public health concern that Member States need to continue to address. Using the criteria of two EU-level nutrient profile models, our study suggests that a sizable share of pre-packaged foods in the categories of breakfast cereals, ready meals, processed meat, processed seafood, and yoghurts, contributes to these excessive intakes in Europe. Consequently, additional improvements to the food supply or consumption habits will be needed to achieve the necessary population diet improvements and meet international goals and targets, such as the voluntary WHO NCD targets [47] and the sustainable development goal 3.4 [48]. Reformulation and restricted marketing of those foods that most contribute the above nutrients to people's (including children's) diets are seen as key measures in the move towards health-promoting food environments. Among others, nutrient profiling helps to provide targets for food product improvement and criteria for checking which products may be deemed suitable for marketing to children. More efforts in monitoring the nutritional composition of the food supply as well as complementary measures, such as fiscal measures, clear nutrition information, marketing restrictions, and public procurement criteria for food can provide additional and strong incentives and a level playing field for producers towards a more healthful food supply overall.

## Supporting information

S1 File **Table A. Number of total and ineligible products in five product categories and for 20 countries from the Euromonitor Nutrition 2016 database using the EU Pledge nutrient profile model.** Total product numbers are the result of extracting all products from the Euromonitor Nutrition 2016 database with complete and plausible nutrition information as well as a non-zero brand share as indicated in the Euromonitor Packaged Foods 2016 database. Number of ineligible products refers to all those products not meeting the category-specific criteria defined in the EU Pledge nutrient profile model. Inter-country comparisons should not be made. **Table B. Number of total and ineligible products in five product categories and for 20 countries from the Euromonitor Nutrition 2016 database using the WHO Europe nutrient profile model.** Total product numbers are the result of extracting all products from the Euromonitor Nutrition 2016 database with complete and plausible nutrition information as well as a non-zero brand share as indicated in the Euromonitor Packaged Foods 2016 database. Number of ineligible products refers to all those products not meeting the category-specific criteria defined in the WHO Europe nutrient profile model. Inter-country comparisons should not be made. **Table C. Assessment of the minimum, median, and maximum difference from applicable nutrient/energy thresholds for breakfast cereals and processed meat products from the Euromonitor Nutrition 2016 database using the EU Pledge nutrient profile model.** Differences from the various applicable criteria thresholds were assessed for all products listed in the Euromonitor Nutrition 2016 database with complete and plausible nutrition information and non-zero brand share as indicated in the Packaged Foods 2016 database. For the upper limit values (total sugars, salt, saturated fat, and energy), the threshold figure was subtracted from the actual figures in the nutrition information. For the lower limit value (protein, fibre), the actual figures in the nutrition information were subtracted from the threshold figure. Resulting values above zero were used to calculate the minimum, median, interquartile range, and maximum differences from the applicable category thresholds. **Table D. Assessment of the minimum, median, and maximum difference from applicable nutrient/energy thresholds for processed seafood products and ready meals from the Euromonitor Nutrition 2016 database using the EU Pledge nutrient profile model.** Differences from the various applicable criteria thresholds were assessed for all products listed in the Euromonitor Nutrition 2016 database with complete and plausible nutrition information and non-zero brand share as indicated in the Packaged Foods 2016 database. For the upper limit values (total sugars, salt, saturated fat, and energy), the threshold figure was subtracted from the actual figures in the nutrition information. For the lower limit value (protein), the actual figures in the nutrition information were subtracted from the threshold figure. Resulting values above zero were used to calculate the minimum, median, interquartile range, and maximum differences from the applicable category thresholds. **Table E. Assessment of the minimum, median, and maximum difference from applicable nutrient/energy thresholds for yoghurt products from the Euromonitor Nutrition 2016 database using the EU Pledge nutrient profile model.** Differences from the various applicable criteria thresholds were assessed for all products listed in the Euromonitor Nutrition 2016 database with complete and plausible nutrition information and non-zero brand share as indicated in the Packaged Foods 2016 database. For the upper limit values (total sugars, salt, saturated fat, and energy), the threshold figure was subtracted from the actual figures in the nutrition information. Resulting values above zero were used to calculate the minimum, median, interquartile range, and maximum differences from the applicable category thresholds.
(DOCX)
