# Supplementary material for: The ineligibility of food products from across the EU for marketing to children according to two EU-level nutrient profile models
Source: PLoS One. 2019 Oct 23;14(10):e0213512. doi: 10.1371/journal.pone.0213512 (PMC6808307; doi:10.1371/journal.pone.0213512)
Supplement: S1 File — Table A. Number of total and ineligible products in five product categories and for 20 countries from the Euromonitor Nutrition 2016 database using the EU Pledge nutrient profile model. Total product numbers are the result of extracting all products from the Euromonitor Nutrition 2016 database with complete and plausible nutrition information as well as a non-zero brand share as indicated in the Euromonitor Packaged Foods 2016 database. Number of ineligible products refers to all those products not meeting the category-specific criteria defined in the EU Pledge nutrient profile model. Inter-country comparisons should not be made. Table B. Number of total and ineligible products in five product categories and for 20 countries from the Euromonitor Nutrition 2016 database using the WHO Europe nutrient profile model. Total product numbers are the result of extracting all products from the Euromonitor Nutrition 2016 database with complete and plausible nutrition information as well as a non-zero brand share as indicated in the Euromonitor Packaged Foods 2016 database. Number of ineligible products refers to all those products not meeting the category-specific criteria defined in the WHO Europe nutrient profile model. Inter-country comparisons should not be made. Table C. Assessment of the minimum, median, and maximum difference from applicable nutrient/energy thresholds for breakfast cereals and processed meat products from the Euromonitor Nutrition 2016 database using the EU Pledge nutrient profile model. Differences from the various applicable criteria thresholds were assessed for all products listed in the Euromonitor Nutrition 2016 database with complete and plausible nutrition information and non-zero brand share as indicated in the Packaged Foods 2016 database. For the upper limit values (total sugars, salt, saturated fat, and energy), the threshold figure was subtracted from the actual figures in the nutrition information. For the lower limit value (protein, fibre [file pone.0213512.s001.docx]

S1 File – Supporting information

S1 Table A. Number of total and ineligible products in five product categories and for 20 countries from the Euromonitor Nutrition 2016 database using the EU Pledge nutrient profile model.

| **Country^a^** | **Breakfast cereals** | | **Processed meat** | | **Processed seafood** | | **Ready meals** | | **Yoghurts** | | **All categories** | |
| --- | --- | --- | --- | --- | --- | --- | --- | --- | --- | --- | --- | --- |
|  | total | ineligible | total | ineligible | total | ineligible | total | ineligible | total | ineligible | total | ineligible |
| AT | 22 | 6 | 34 | 21 | 24 | 11 | 34 | 25 | 25 | 8 | 139 | 71 |
| BE | 42 | 14 | 26 | 14 | 13 | 8 | 43 | 28 | 35 | 10 | 159 | 74 |
| BG | 20 | 9 | 8 | 7 | 3 | 3 | 4 | 4 | 17 | 4 | 52 | 27 |
| CZ | 37 | 21 | 12 | 8 | 11 | 5 | 18 | 14 | 47 | 23 | 125 | 71 |
| DK | 35 | 12 | 22 | 17 | 15 | 11 | 11 | 2 | 13 | 0 | 96 | 42 |
| FI | 39 | 14 | 42 | 13 | 11 | 5 | 30 | 17 | 49 | 10 | 171 | 59 |
| FR | 41 | 13 | 32 | 22 | 20 | 13 | 36 | 15 | 30 | 2 | 159 | 65 |
| DE | 37 | 16 | 30 | 20 | 17 | 8 | 42 | 29 | 42 | 18 | 168 | 91 |
| GR | 27 | 8 | 11 | 9 | 11 | 5 | 32 | 22 | 37 | 14 | 118 | 58 |
| HU | 45 | 26 | 31 | 29 | 15 | 12 | 11 | 10 | 44 | 15 | 146 | 92 |
| IE | 53 | 10 | 11 | 7 | 9 | 7 | 19 | 9 | 38 | 12 | 130 | 45 |
| IT | 26 | 9 | 38 | 26 | 27 | 18 | 43 | 32 | 51 | 13 | 185 | 98 |
| NL | 22 | 6 | 26 | 14 | 16 | 2 | 23 | 14 | 38 | 9 | 125 | 45 |
| PL | 48 | 19 | 7 | 4 | 12 | 7 | 23 | 17 | 32 | 11 | 122 | 58 |
| PT | 40 | 18 | 20 | 13 | 18 | 11 | 20 | 15 | 36 | 5 | 134 | 62 |
| RO | 19 | 9 | 2 | 2 | 1 | 1 | 4 | 1 | 17 | 2 | 43 | 15 |
| SK | 33 | 17 | 6 | 6 | 10 | 7 | 18 | 13 | 29 | 12 | 96 | 55 |
| ES | 27 | 11 | 24 | 14 | 26 | 19 | 32 | 23 | 35 | 11 | 144 | 78 |
| SE | 51 | 16 | 51 | 41 | 29 | 23 | 28 | 20 | 20 | 3 | 179 | 103 |
| GB | 57 | 13 | 37 | 19 | 20 | 7 | 47 | 19 | 39 | 14 | 200 | 72 |
| Column total | **721** | **267** | **470** | **306** | **308** | **183** | **518** | **329** | **674** | **196** | **2691** | **1281** |

Total product numbers are the result of extracting all products from the Euromonitor Nutrition 2016 database with complete and plausible nutrition information as well as a non-zero brand share as indicated in the Euromonitor Packaged Foods 2016 database. Number of ineligible products refers to all those products not meeting the category-specific criteria defined in the EU Pledge nutrient profile model. ^a^ ISO 3166-1 alpha-2 country code. Inter-country comparisons should not be made.

S1 Table B. Number of total and ineligible products in five product categories and for 20 countries from the Euromonitor Nutrition 2016 database using the WHO Europe nutrient profile model.

| **Country^a^** | **Breakfast cereals** | | **Processed meat** | | **Processed seafood** | | **Ready meals** | | **Yoghurts** | | **All categories** | |
| --- | --- | --- | --- | --- | --- | --- | --- | --- | --- | --- | --- | --- |
|  | total | ineligible | total | ineligible | total | ineligible | total | ineligible | total | ineligible | total | ineligible |
| AT | 22 | 19 | 34 | 19 | 24 | 5 | 34 | 26 | 25 | 19 | 139 | 88 |
| BE | 42 | 33 | 26 | 14 | 13 | 5 | 43 | 27 | 35 | 27 | 159 | 106 |
| BG | 20 | 17 | 8 | 7 | 3 | 2 | 4 | 4 | 17 | 14 | 52 | 44 |
| CZ | 37 | 33 | 12 | 9 | 11 | 3 | 18 | 14 | 47 | 47 | 125 | 106 |
| DK | 35 | 18 | 22 | 16 | 15 | 3 | 11 | 2 | 13 | 8 | 96 | 47 |
| FI | 39 | 24 | 42 | 16 | 11 | 3 | 30 | 16 | 49 | 31 | 171 | 90 |
| FR | 41 | 35 | 32 | 20 | 20 | 6 | 36 | 17 | 30 | 20 | 159 | 98 |
| DE | 37 | 34 | 30 | 21 | 17 | 6 | 42 | 29 | 42 | 39 | 168 | 129 |
| GR | 27 | 23 | 11 | 10 | 11 | 3 | 32 | 24 | 37 | 28 | 118 | 88 |
| HU | 45 | 43 | 31 | 28 | 15 | 5 | 11 | 10 | 44 | 37 | 146 | 123 |
| IE | 53 | 39 | 11 | 6 | 9 | 2 | 19 | 9 | 38 | 24 | 130 | 80 |
| IT | 26 | 24 | 38 | 26 | 27 | 12 | 43 | 33 | 51 | 43 | 185 | 138 |
| NL | 22 | 17 | 26 | 16 | 16 | 3 | 23 | 12 | 38 | 22 | 125 | 70 |
| PL | 48 | 40 | 7 | 7 | 12 | 3 | 23 | 16 | 32 | 26 | 122 | 92 |
| PT | 40 | 38 | 20 | 12 | 18 | 3 | 20 | 15 | 36 | 21 | 134 | 89 |
| RO | 19 | 16 | 2 | 1 | 1 | 1 | 4 | 1 | 17 | 13 | 43 | 32 |
| SK | 33 | 30 | 6 | 6 | 10 | 2 | 18 | 14 | 29 | 27 | 96 | 79 |
| ES | 27 | 24 | 24 | 17 | 26 | 11 | 32 | 27 | 35 | 23 | 144 | 102 |
| SE | 51 | 26 | 51 | 38 | 29 | 17 | 28 | 20 | 20 | 13 | 179 | 114 |
| GB | 57 | 41 | 37 | 15 | 20 | 1 | 47 | 18 | 39 | 32 | 200 | 107 |
| Column total | **721** | **574** | **470** | **304** | **308** | **96** | **518** | **334** | **674** | **514** | **2691** | **1822** |

Total product numbers are the result of extracting all products from the Euromonitor Nutrition 2016 database with complete and plausible nutrition information as well as a non-zero brand share as indicated in the Euromonitor Packaged Foods 2016 database. Number of ineligible products refers to all those products not meeting the category-specific criteria defined in the WHO Europe nutrient profile model. ^a^ ISO 3166-1 alpha-2 country code. Inter-country comparisons should not be made.

S1 Table C. Assessment of the minimum, median, and maximum difference from applicable nutrient/energy thresholds for breakfast cereals and processed meat products from the Euromonitor Nutrition 2016 database using the EU Pledge nutrient profile model.

|  | Breakfast cereals (721 products) | | | | | Processed meat (470 products) | | | | | |
| --- | --- | --- | --- | --- | --- | --- | --- | --- | --- | --- | --- |
| Criterion | Total sugars [g] | Salt [g] | Sat fat [g] | Fibre [g] | Energy [kcal] | Salt [g] | Sat fat [g] | Total sugars [g] | Protein [g] | Energy [kcal] |  |
| Threshold per 100 g | ≤30 | ≤1.125 | ≤5 | ≥3 | ≤567 | ≤2 | ≤6 | ≤5 | ≥12 en% | ≤378 |  |
| No. of products not meeting threshold | 86 | 104 | 57 | 100 | 0 | 166 | 172 | 12 | 15 | 18 |  |
| % of products not meeting threshold | 12% | 14% | 8% | 14% | - | 35% | 37% | 3% | 3% | 4% |  |
| Minimum difference from threshold | 0.1 | 0.1 | 0.1 | 0.1 | - | 0.1 | 0.1 | 0.1 | 0.1 | 3 |  |
| Maximum difference from threshold | 14.4 | 1.7 | 7.5 | 3.0 | - | 4.0 | 18.7 | 32.0 | 9.4 | 184 |  |
| Median | 5.0 | 0.7 | 1.6 | 1.0 | - | 0.5 | 2.5 | 1.9 | 1.3 | 112 |  |
| Interquartile range | 3.2-7.0 | 0.2-0.9 | 0.8-3.0 | 0.5-1.0 | - | 0.3-1.4 | 1.2-4.0 | 0.4-7.0 | 0.2-2.7 | 71-150 |  |

Differences from the various applicable criteria thresholds were assessed for all products listed in the Euromonitor Nutrition 2016 database with complete and plausible nutrition information and non-zero brand share as indicated in the Packaged Foods 2016 database. For the upper limit values (total sugars, salt, saturated fat, and energy), the threshold figure was subtracted from the actual figures in the nutrition information. For the lower limit value (protein, fibre), the actual figures in the nutrition information were subtracted from the threshold figure. Resulting values above zero were used to calculate the minimum, median, interquartile range, and maximum differences from the applicable category thresholds. Abbreviations: Sat fat, saturated fat; n/a, not applicable.

S1 Table D. Assessment of the minimum, median, and maximum difference from applicable nutrient/energy thresholds for processed seafood products and ready meals from the Euromonitor Nutrition 2016 database using the EU Pledge nutrient profile model.

|  | Processed seafood (308 products) | | | | | Ready meals (518 products) | | | |
| --- | --- | --- | --- | --- | --- | --- | --- | --- | --- |
| Criterion | Salt [g] | Sat fat [g] | Total sugars [g] | Protein [g] | Energy [kcal] | Sat Fat [g] | Total sugars [g] | Salt [g] | Energy [kcal] |
| Threshold per 100 g | ≤2 | ≤33% of total fat | ≤5 | ≥12 en% | ≤378 | ≤5 | ≤7.5 | ≤1 | ≤212.5 |
| No. of products not meeting threshold | 68 | 41 | 25 | 22 | 16 | 44 | 27 | 268 | 210 |
| % of products not meeting threshold | 22% | 13% | 8% | 7% | 5% | 8% | 5% | 52% | 41% |
| Minimum difference from threshold | 0.1 | 0.0 | 0.3 | 0.3 | 0 | 0.1 | 0.1 | 0.1 | 1 |
| Maximum difference from threshold | 13.9 | 4.6 | 21.5 | 13.8 | 175 | 7.0 | 8.5 | 12.0 | 318 |
| Median | 1.0 | 0.1 | 7.8 | 2.2 | 38 | 1.1 | 1.7 | 0.4 | 43 |
| Interquartile range | 0.5-2.5 | 0.0-0.7 | 2.8-12.8 | 0.9-5.0 | 49-94 | 0.3-2.1 | 0.6-3.5 | 0.2-0.7 | 20-88 |

Differences from the various applicable criteria thresholds were assessed for all products listed in the Euromonitor Nutrition 2016 database with complete and plausible nutrition information and non-zero brand share as indicated in the Packaged Foods 2016 database. For the upper limit values (total sugars, salt, saturated fat, and energy), the threshold figure was subtracted from the actual figures in the nutrition information. For the lower limit value (protein), the actual figures in the nutrition information were subtracted from the threshold figure. Resulting values above zero were used to calculate the minimum, median, interquartile range, and maximum differences from the applicable category thresholds. Abbreviations: Sat fat, saturated fat; n/a, not applicable.

S1 Table E. Assessment of the minimum, median, and maximum difference from applicable nutrient/energy thresholds for yoghurt products from the Euromonitor Nutrition 2016 database using the EU Pledge nutrient profile model.

|  | Yoghurts (674 products) | | | |
| --- | --- | --- | --- | --- |
| Criterion | Sat fat [g] | Total sugars [g] | Salt [g] | Energy [kcal] |
| Threshold per 100 g | ≤2.6 | ≤13.5 | ≤0.75 | ≤113 |
| No. of products not meeting threshold | 86 | 142 | 1 | 82 |
| % of products not meeting threshold | 13% | 21% | 0% | 12% |
| Minimum difference from threshold | 0.1 | 0.1 | 0.55 | 0 |
| Maximum difference from threshold | 4.5 | 14.9 | 0.55 | 62 |
| Median | 2.4 | 1.2 | n/a | 16 |
| Interquartile range | 0.7-3.1 | 0.5-2.0 | n/a | 7-28 |

Differences from the various applicable criteria thresholds were assessed for all products listed in the Euromonitor Nutrition 2016 database with complete and plausible nutrition information and non-zero brand share as indicated in the Packaged Foods 2016 database. For the upper limit values (total sugars, salt, saturated fat, and energy), the threshold figure was subtracted from the actual figures in the nutrition information. Resulting values above zero were used to calculate the minimum, median, interquartile range, and maximum differences from the applicable category thresholds. Abbreviations: Sat fat, saturated fat; n/a, not applicable.
